# Hereditary breast cancer: emerging roles of non-coding RNAs

**DOI:** 10.1186/s41065-026-00720-8

**Published:** 2026-09-28

**Authors:** Zhenshan Yang, Dipanjan Guha, Ling Zhang, Honglu Diao, Angshuman Bagchi

**Affiliations:** 1 Reproductive Medicine Center, Biomedical Engineering College, Renmin Hospital, Hubei University of Medicine, Shiyan, Hubei 442000 P.R. China; 2 Hubei Clinical Research Center for Reproductive Medicine, Shiyan, Hubei 442000 P.R. China; 3 Shiyan Key Laboratory of Reproduction and Genetics, Renmin Hospital, Hubei University of Medicine, Shiyan, Hubei 442000 P.R. China; 4 Hubei Key Laboratory of Embryonic Stem Cell Research, Hubei University of Medicine, Shiyan, Hubei 442000 P.R. China; 5 DBT-funded Bioinformatics Infrastructure Facility, University of Kalyani, Kalyani-741235, Nadia, West Bengal India; 6 Hubei University of Medicine, Library, Shiyan, Hubei 442000 P.R. China; 7 Biomedical Research Institute, Hubei University of Medicine, Shiyan, Hubei 442000 P.R. China; 8 Department of Biochemistry and Biophysics, University of Kalyani, Kalyani-741235, Nadia, West Bengal India

**Keywords:** Breast cancer, Heredity, Non-coding RNAs, Treatment strategies

## Abstract

Breast cancer remains a major global health burden and is characterized by substantial molecular and clinical heterogeneity. In recent years, considerable progress has been made in elucidating the genetic and molecular mechanisms underlying breast cancer, particularly those associated with hereditary susceptibility. Advances in genomic and functional studies have further revealed the complex mutational landscape of breast cancer, including key alterations in tumor suppressor genes, oncogenic signaling pathways, and mutagenic processes. In addition, non-coding RNAs (ncRNAs) have emerged as important regulators of gene expression, influencing tumor progression and therapeutic resistance. Emerging strategies, including immunotherapy, precision medicine, and nanoparticle-based drug delivery systems, have significantly expanded treatment options and improved clinical outcomes. However, challenges such as tumor heterogeneity, drug resistance, systemic toxicity, and disparities in healthcare access continue to limit the effectiveness and broad implementation of these therapies. This review provides a comprehensive overview of the molecular mechanisms of breast cancer from a hereditary perspective, with a particular focus on genetic susceptibility, ncRNAs, and emerging therapeutic strategies. Additionally, it highlights current limitations and future directions, emphasizing the need for integrative approaches that combine genomic insights, advanced technologies, and personalized medicine to improve breast cancer prevention and treatment.

## Background

Breast cancer remains one of the most common malignancies worldwide, predominantly affecting women. In 2022 alone, it was diagnosed in approximately 2.3 million individuals and resulted in 670,000 deaths [1, 2]. Although its occurrence is strongly associated with gender, both incidence and mortality are also influenced by factors such as age, ethnicity, geographic location, and access to healthcare. For instance, women of African descent generally exhibit lower incidence rates but higher mortality compared to Caucasian women. This disparity is often linked to differences in access to healthcare services and treatment options [3, 4]. Global comparisons further reveal that high-income countries report higher incidence rates than low-income regions, likely due to more comprehensive screening programs that facilitate early detection. In contrast, mortality rates are disproportionately higher in low-income countries, reflecting limited access to screening and adequate medical care [5–7].

The heterogeneity of breast cancer presents a major challenge for the development of effective and targeted therapies. Molecular profiling has enabled the classification of breast cancer into four principal subtypes: luminal A (ER + PR+HER2-), luminal B (ER+, PR+, HER2+), HER2-positive (ER-PR-HER2+), and triple-negative (ER-PR-HER2-) breast cancer [8–10].

**Fig. 1:**
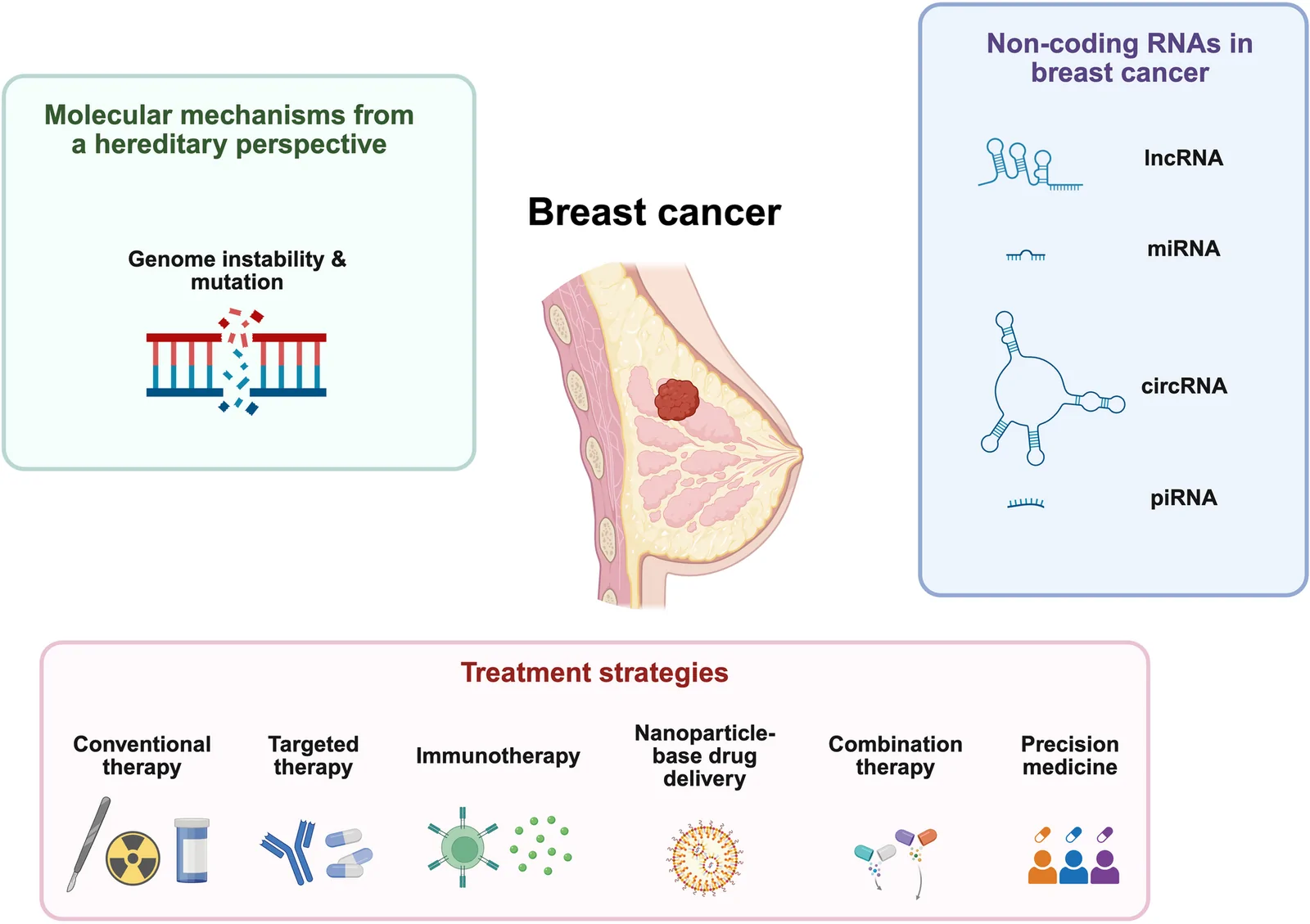
Schematic representation of the hereditary and molecular landscape of breast cancer and associated therapeutic strategies

Breast cancer can be categorized into two types based on its development: hereditary (familial) and sporadic. Genetic predisposition plays a critical role in breast cancer susceptibility. While high-penetrance mutations in genes such as BRCA1 and BRCA2 confer substantial risk, they account for only a minority of cases [11, 12]. In addition, non-coding RNAs (ncRNAs) have gained increasing attention as regulators of gene expression and contributors to tumor biology. Despite significant therapeutic advances, including targeted therapies, immunotherapy, and combination therapy, major challenges remain (Fig. 1). Tumor heterogeneity, drug resistance, immune evasion, and treatment-related toxicity continue to limit long-term clinical outcomes. A deeper understanding of the molecular mechanisms underlying breast cancer is essential for improving risk prediction and developing more effective treatment strategies.

## Overview of the latest information on the molecular mechanism of breast cancer onset from a hereditary perspective

Approximately 5–10% of breast cancer cases are attributed to hereditary factors. Among these, mutations in the *BRCA* genes represent the most well-known and prevalent genetic drivers [13]. In particular, *BRCA1* and *BRCA2* mutations, nearly 60% and 45%, respectively, will develop breast cancer by age 70 [14]. In addition to *BRCA1* and *BRCA2*, a growing number of susceptibility genes have been identified as important contributors to hereditary breast cancer risk, including *BARD1*, *STK11*, *RAD51C*, *RAD51D*, *PALB2*, *TP53*, *PTEN*, *CDH1*, *MSH1*, *MSH6*, *MLH1*, *ATM*, and *CHEK2* [15–18]. Loss-of-function mutations in PALB2 significantly contribute to hereditary breast cancer. The lifetime risk of breast cancer for female PALB2 mutation carriers by age 70 varies from 33% in women with no family history to 58% in those with two or more first-degree relatives with breast cancer at age 50 [19]. Quantitative findings indicate that greater CHK2 kinase dysfunction is linked to a higher risk of breast cancer [20]. Individuals with pathogenic variants in *ATM* have a 2- to 4-fold higher risk of developing breast cancer [21]. Women with a pathogenic *TP53* variant face an 80–90% lifetime risk of developing breast cancer. Genetic variants are not only crucial in initiating breast tumors but also influence various other stages in the multistep process of breast cancer development. Despite these advances, the majority of breast cancer cases (approximately 90%) arise from acquired (non-inherited) genetic and epigenetic alterations, many of which remain incompletely characterized [22]. This highlights that significant gaps persist in our understanding of breast cancer etiology and underscores the need for continued investigation into its molecular mechanisms.

Genetic mutations and dysregulation of signaling pathways play central roles in the initiation and progression of breast cancer. These alterations can impair critical cellular processes, including cell growth, proliferation, survival, apoptosis, and DNA repair, thereby contributing to tumorigenesis [23]. Recent studies have identified novel germline variants associated with disease progression. For example, a common missense germline variant in proprotein convertase subtilisin/kexin type 9 (PCSK9, rs562556, V474I) has been linked to reduced survival in patients with breast cancer. Further research indicated that a germline variant of PCSK9 promotes breast cancer metastasis via targeting the LRP1 receptor [24]. Functional modeling of thisgain-of-function single-nucleotide variant in murine systems further demonstrated its causal role in promoting breast cancer metastasis. Li et al. suggested that epigenetic alteration effect BRCA1 haploinsufficiency promotes early tumor onset in breast cancer. Transcription factor analyses identify loss of ELF5 and gain of AP-1 sites in these epigenetically primed regions; in vivo experiments further implicate AP-1 and Wnt10a as strong promoters of BRCA1-related breast cancer [25].

Genetic predisposition plays a major role in breast cancer risk. While high-penetrance pathogenic variants such as BRCA1/2 confer a high risk, they account for only a small proportion of cases [11, 12]. Yao et al. provided a high-resolution mutational landscape of triple-negative breast cancer (TNBC) in African American women. Their analysis revealed that the genomic profile is predominantly characterized by alterations in *TP53*, *NOTCH1*, *RB1*, *KMT2D*, *PIK3CA*, *PTEN*, *KMT2C*, *BRCA1*, and *NF1*. Notably, no evidence of associations of mutational features with African ancestry [26]. Genome-wide association studies (GWAS) have identified hundreds of genetic susceptibility loci associated with breast cancer risk. However, the majority of causal variants and their corresponding target genes remain insufficiently characterized. Recent large-scale fine-mapping efforts across multiple ancestries have substantially improved the resolution of breast cancer risk loci, revealing 332 independent association signals and candidate susceptibility genes. Integrative analyses combining functional genomics, single-cell transcriptomics, and experimental validation have implicated multiple oncogenic pathways, such as PI3K/AKT, TNF/NF-κB, p53, and Wnt/β-catenin signaling, that might mediate breast cancer risk [27].

Polygenic risk scores (PRS), which integrate the cumulative effects of numerous common genetic variants, have emerged as important tools for evaluating individual susceptibility to breast cancer [28]. PRS captures much of the variation in predisposition by aggregating the effects of hundreds of thousands of variants discovered by GWAS. Therefore, PRS could stratify risk to guide screening and support earlier disease prediction and preventive strategies [29, 30]. Recent studies have further demonstrated that PRS models can effectively predict breast cancer risk across diverse populations, including women of European, Asian, and African ancestry [30–33]. Future studies integrating PRS, germline genetic testing, and genetic counseling may further improve individualized risk prediction and precision management of hereditary breast cancer.

Drug resistance remains a major challenge in breast cancer management and represents a leading cause of cancer-related mortality in patients [34]. Acquired genetic alterations play a critical role in the development of resistance to endocrine and targeted therapies, particularly in metastatic breast cancer. Recent evidence has highlighted mutagenesis of apolipoprotein B mRNA-editing enzyme catalytic polypeptide-like 3 (APOBEC3) as a key mechanism underlying therapeutic resistance. APOBEC3-associated mutational signatures are frequently enriched in post-treatment hormone receptor-positive breast cancers and have been linked to poorer progression-free survival in patients receiving endocrine therapy in combination with CDK4/6 inhibitors [35]. Further research suggests that active APOBEC3 mutagenesis contributes to therapy resistance by causing RB1 loss.

Currently, much of the fundamental research on breast cancer has been translated into clinical applications. Comprehensive genetic and genomic test has become an essential component of precision medicine in breast cancer by identifying molecular alterations, and enabling personalized therapeutic strategies. Genetic testing in clinical settings typically involves a blood test, a saliva sample, or a cell swab. It helps identify gene mutations associated with an increased risk of breast cancer. Genomic tests (e.g., Breast Cancer Index test and MammaPrint test) analyze genes in a breast tumor to predict the chances of cancer growth, spread, or recurrence after treatment. They also assist doctors in choosing the most effective targeted therapies for a particular breast cancer. In addition, many new targeted therapies have emerged clinically and are now being quickly integrated into routine care [36].

In addition to recent advances in genomic studies, several clinical practice guidelines have further refined the risk assessment and management strategies for hereditary breast cancer. The NCCN Guidelines for Genetic/Familial High-Risk Assessment: Breast, Ovarian, and Pancreatic mainly concentrate on evaluating pathogenic or likely pathogenic (P/LP) variants linked to a higher risk of breast cancer [37]. The ASCO-Society of Surgical Oncology Guideline provides recommendations for germline mutation testing in breast cancer patients [38]. The ESMO Clinical Practice Guideline offers recommendations for risk reduction and screening in individuals with hereditary breast and ovarian cancer syndromes [39].

## Roles of ncRNAs as mediators of breast cancers

**Fig. 2:**
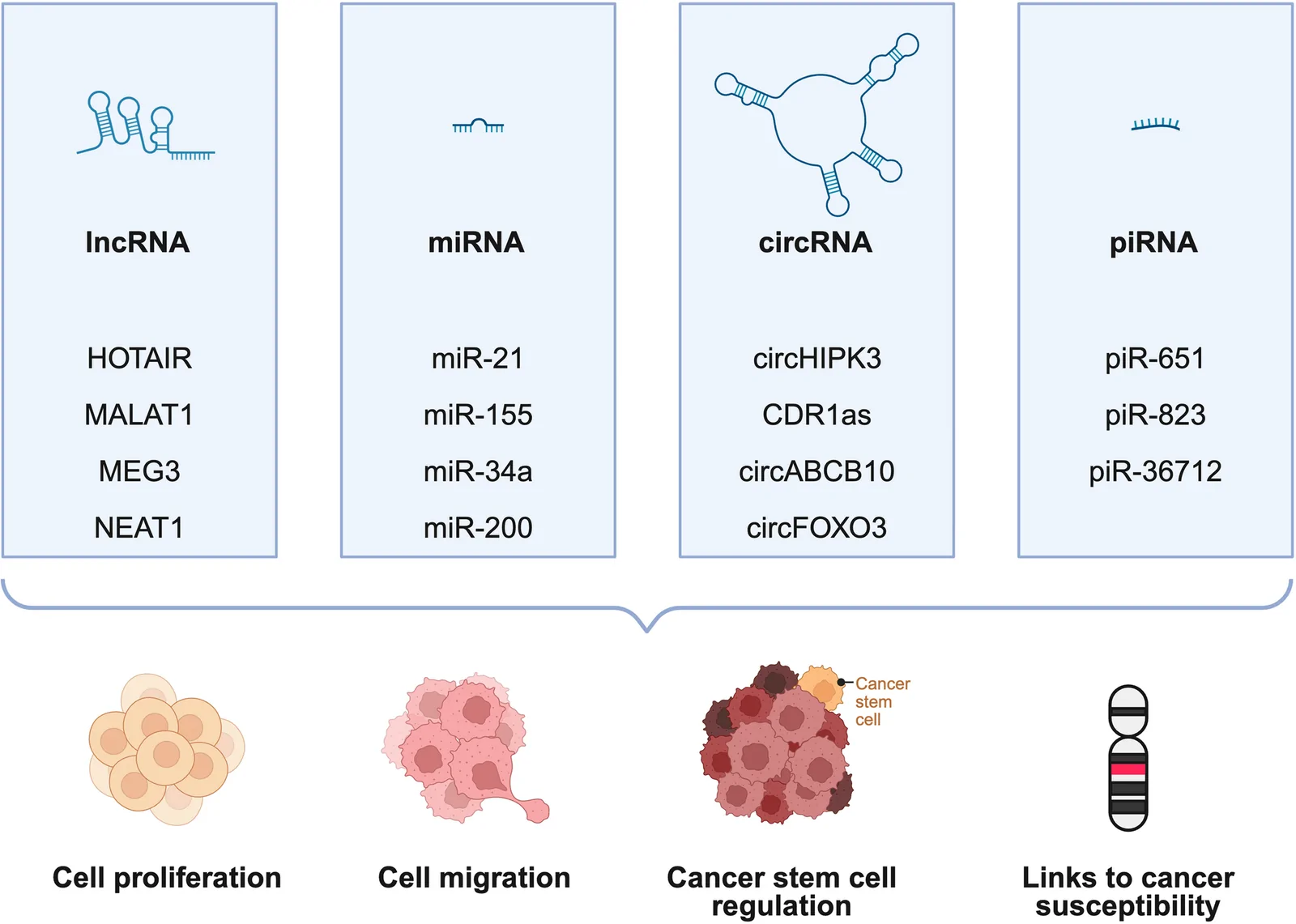
Significant ncRNAs in regulating breast cancer

NcRNAs are special classes of RNA molecules; most of them do not encode proteins but possess a significant role in the regulation of gene expression at both transcriptional and post-transcriptional stages. The main categories of ncRNAs include long non-coding RNAs (lncRNAs), microRNAs (miRNAs), circular RNAs (circRNAs), Piwi-interacting RNAs (piRNAs), etc [40, 41]. These molecules are important for controlling cell proliferation and metastasis, they eventually play a vital role in controlling breast cancer and proved to be potent biomarkers summarized in Table 1 and in Fig. 2. ncRNAs also play an important role in hereditary breast cancer syndromes [42–44]. Tanic et al. identified miRNAs associated with hereditary breast cancer and miR-30c regulating the KRAS oncogene [45]. It has been showed that single variant in Pri-miRNA-155 linked to hereditary breast cancer susceptibility increases aggressiveness in cancer cells [46].

**Table 1:** NcRNAs as clinical biomarkers in breast cancer

Types	Name	Major Biological Role	Clinical Biomarker Types	References
LncRNAs	HOTAIR	Predictor of metastasis	Prognostic	[47]
	MALAT1	Regulates cell proliferation, migration, and invasion	Prognostic	[48, 49]
	MEG3	Tumor suppressor that activates p53 signaling	Prognostic	[50, 51]
	NEAT1	Promotes cell proliferation, migration, and metastasis	Prognostic	[52, 53]
MiRNAs	miR-21	Promotes tumor growth by inhibiting tumor suppressor genes	Diagnostic and prognostic	[54, 55]
	miR-155	Suppresses tumor progression via enhancing immune cell recruitment	Prognostic	[56]
	miR-34a	Overcomes acquired drug resistance and disease progression	Diagnostic and prognostic	[57]
	miR-200	Suppresses cancer cell migration.	Diagnostic and prognostic	[58, 59]
CircRNAs	circHIPK3	Promotes cell proliferation	Diagnostic	[60, 61]
	CDR1as	Increase the resistance of breast cancer cells to cisplatin	Prognostic	[62, 63]
	circABCB10	Increases cell proliferation	Diagnostic	[64, 65]
	circFOXO3	Inhibits cancer growth and metastasis	Prognostic	[66]
PiRNAs	piR-651	Enhances survival and metastasis of cancer cells	-	[67, 68]
	piR-823	Enhances proliferation and involves in cancer stem cell regulation	-	[67, 69]
	piR-36,712	Ihibits cancer cell proliferation	Prognostic	[70]

## LncRNAs and breast cancers

LncRNAs belong to a class of ncRNA molecules, which are longer than 200 nucleotides. Most of them do not encode proteins, but still have potency in gene expression regulation. They function at various levels, including chromatin remodeling, transcriptional, and post-transcriptional control. These groups of ncRNA molecules can act as molecular scaffolds, guides, or signals, thereby influencing cellular homeostasis [71].

In breast cancer, lncRNAs are significantly involved in the initiation of the tumor, progression, and metastasis. HOTAIR is one of the most well-studied oncogenic lncRNAs. It plays a crucial role in breast cancer by contributing to its progression and serving as a predictor of metastasis [47, 72]. Similarly, MALAT1 is responsible for the enhanced cell proliferation and poor prognosis in breast cancer patients [48, 49]. On the other hand, some lncRNAs act as tumor suppressors. For example, MEG3 regulates the p53 pathway and suppresses tumor growth [50]. LncRNAs also interact with microRNAs by acting as competing endogenous RNAs, effectively “sponging” miRNAs and disallowing them to bind target mRNAs [73, 74]. This interaction influences key signaling pathways, e.g., PI3K/AKT, Wnt/β-catenin, and estrogen receptor signaling, all of which are crucial in breast cancer biology. Clinically, lncRNAs show great potential as diagnostic and prognostic biomarkers due to their consistent tissue-specific expression.

### MiRNAs and breast cancers

MiRNAs are small non-coding RNA molecules, approximately 20–24 nucleotides in length, that regulate gene expression by binding to messenger RNAs (mRNAs) and either inhibiting translation or promoting mRNA degradation [75, 76] Several miRNAs have been identified as being involved in breast cancer progression. For example, miR-21 is an oncogenic miRNA that promotes tumor growth by inhibiting tumor suppressor genes such as PTEN [54]. In contrast, miR-155 is associated with immunue cell recruitment and anti-tumor behavior [56]. MiR-34a can induce apoptosis and cell cycle arrest, and thereby functions as a tumor suppressor. It can also overcome drug resistance in breast cancers [57]. miR-200 can inhibit metastasis by reducing epithelial-mesenchymal transition [58, 77]. Similarly, let-7 family miRNAs can modulate the proliferation and differentiation of breast cancer cells and can act as tumor suppressors [78]. miR-125b is multifaceted, capable of acting as either a tumor suppressor or an oncogene, depending on the cellular context [79, 80]. Overall, miRNAs serve as important biomarkers of breast cancer, offering promising avenues for personalized medicine and improved treatment outcomes.

### CircRNAs and breast cancers

CircRNAs are endogenous ncRNAs. They possess a covalently closed-loop structure without having 5’ caps or 3’ poly(A) tails. They are generated through back-splicing, in which a downstream splice donor is connected to an upstream splice acceptor. This covalently closed structure confers remarkable stability to circRNAs, rendering them resistant to exonuclease-mediated degradation and enabling their accumulation in cells and body fluids [81].

In breast cancer, circRNAs play a significant role in tumor progression. One of their primary mechanisms is acting as “miRNA sponges”. By binding to miRNAs, circRNAs sequester them and reduce their inhibitory effects on target mRNAs, thereby regulating gene expression. Additionally, peptides encoded by circRNA influence the activation of transcription factors, which in turn regulate the transcription of target genes [82]. Several circRNAs have been implicated in breast cancer. For example, circHIPK3 promotes cell proliferation and tumor growth by sponging miRNAs [60]. CDR1as (ciRS-7) interacts with miR-7, influencing oncogenic signaling pathways [62]. CircABCB10 is associated with increased cell proliferation through sponging miR-1271 [64]. CircFOXO3, in contrast, can act as a tumor suppressor by promoting cell apoptosis [83]. CircRNAs are also involved in key breast cancer-related pathways, including PI3K/AKT, Wnt/β-catenin, and MAPK signaling. They contribute to important processes such as proliferation, migration, invasion, metastasis, angiogenesis, chemoresistance, and endocrine resistance. Due to their high stability, tissue specificity, and presence in exosomes and circulating blood, circRNAs have emerged as promising biomarkers for early diagnosis and disease monitoring.

### PiRNAs and breast cancers

PiRNAs are ncRNAs, with lengths between 26 and 31 nucleotides, which interact with PIWI proteins to regulate gene expression and maintain genomic integrity [84, 85]. In breast cancer, piRNAs are increasingly recognized for their roles in tumorigenesis and progression. For example, inhibition of piR-651 and piR-823 decreased the survival and metastasis of cancer cells [67]. The level of piR-36,712 is downregulated in breast cancer and associated with poor prognosis, where downregulation of piRNA-36,712 increased SEPW1 expression and affected the p53 and Slug expression to promote tumor progression [70]. These findings suggest that piRNAs may serve as potential biomarkers and therapeutic targets in breast cancer.

## Treatment strategies for breast cancer for good health and well-being

### Treatment strategies

Breast cancer is a highly heterogeneous disease encompassing multiple subtypes and stages [86]. There are different treatment strategies for breast cancer. Conventional treatment modalities primarily include surgery, radiotherapy, and chemotherapy. One of the major challenges in these treatment strategies is the heterogeneity in cancer stage and genetic background among patients. In recent years, treatment strategies have increasingly shifted toward receptor-targeted therapies, particularly those directed against HER2 [87]. Despite their clinical benefits, these targeted approaches are frequently associated with significant adverse effects and cause therapeutic resistance [88].

Nowadays, ncRNAs are increasingly recognized as important regulators of diverse biological processes, and their involvement in cancer initiation and progression has been widely reported. As a result, ncRNA-based therapeutic strategies have emerged as promising approaches for breast cancer treatment [89, 90]. One promising therapeutic approach involves using miRNA mimics or inhibitors to restore dysregulated gene expression in cancer [91].

Recent advances in breast cancer therapy have led to the development of several innovative treatment strategies, including antibody-drug conjugates (ADCs), precision medicine, immunotherapy, nanoparticle-based drug delivery systems, and combination therapy [92–96]. ADCs have emerged as a transformative therapeutic approach, enabling the targeted delivery of highly potent cytotoxic agents to tumor cells while minimizing systemic toxicity [97, 98]. Meanwhile, precision medicine approaches guided by genomic and molecular profiling have enabled more personalized treatment strategies, tailoring therapies to individual tumor characteristics and resistance mechanisms [99]. Germline BRCA1/2 mutations are associated with homologous recombination deficiency, creating a therapeutic vulnerability that can be exploited by PARP inhibitors [100]. Clinical studies have demonstrated improved outcomes with agents such as Olaparib and Talazoparib in breast cancer patients with BRCA mutations. Immunotherapy, particularly immune checkpoint inhibitors (PD-L1 and CTLA-4 blockade), has demonstrated promising clinical efficacy in selected subtypes such as TNBC by enhancing anti-tumor immune responses [101]. In parallel, nanoparticle-based platforms have gained increasing attention for their ability to enhance drug bioavailability, tumor targeting, and drug release [102, 103]. Furthermore, combination therapies have improved treatment efficacy by simultaneously targeting multiple pathways involved in cancer cell survival and metabolism [104]. Collectively, these emerging therapeutic strategies have contributed to improved clinical outcomes, including prolonged survival and better quality of life for patients.

### Limitations

There are several challenges in the clinical translation of ncRNA-based therapies for breast cancer even with the therapeutic promise. Most importantly, efficient and targeted delivery remains a major challenge, as naked ncRNAs are rapidly degraded by nucleases, exhibit poor cellular uptake and are rapidly cleared from circulation. Lipid nanoparticles, viral vectors, exosomes and polymer-based carriers improve delivery but may cause immunogenicity, limited tumor specificity or manufacturing challenges. The instability of ncRNA also hampers the therapeutic efficacy, which needs chemical modifications that can change the biological activity. Another challenge is the off-target effects, since one miRNA or other ncRNA can target multiple genes and signaling pathways, leading to unintended gene modulation and adverse biological effects. Other challenges for clinical use include tumor heterogeneity, and the absence of standardized dosing and long-term safety data. Addressing these limitations is essential for successful translation of ncRNA therapeutics into routine breast cancer management.

Despite the promising therapeutic potential of targeted therapy, immunotherapy, nanoparticle-based drug delivery, combination therapy, and precision medicine, several challenges remain before their widespread clinical implementation. Acquired resistance has emerged as a significant obstacle. In addition, optimal patient stratification based on molecular subtype, homologous recombination deficiency status, and biomarker profiles is essential to maximize therapeutic benefit. Finally, long-term efficacy, safety, and quality-of-life outcomes require further evaluation through large prospective clinical trials.

## Conclusions and future perspectives

While recent therapeutic advances have reshaped breast cancer treatment, a multidisciplinary approach that includes genomics, immunology, nanotechnology, ncRNA-based therapies, and bioinformatics will be essential to overcoming current challenges and improving patient outcomes.

Despite substantial progress in breast cancer therapy, several critical challenges remain unresolved. Tumor heterogeneity, drug resistance, immune evasion, and systemic toxicity continue to limit long-term therapeutic efficacy. Epigenetic inheritance and gene-environment interactions play important roles in regulating hereditary breast cancer risk. The ever-changing landscape of breast cancer, driven by genetic and epigenetic changes, requires ongoing adjustment of treatment approaches [86].

In addition, socioeconomic factors, including high treatment costs and limited accessibility, pose significant barriers to the broader clinical implementation of therapies. Addressing these issues will be essential for translating scientific advances into real-world patient benefits. Future research should prioritize optimizing combination therapies and identifying robust, clinically actionable biomarkers for precise patient stratification. Moreover, integrating artificial intelligence and machine learning into precision breast oncology frameworks may enhance risk prediction, treatment selection, and dynamic disease monitoring.

NcRNAs, e.g., miRNAs, lncRNAs, circRNAs, piRNAs, etc., have come up as vital regulators in cancer biology. They influence gene expression, signaling pathways, and key cellular activities. Their dysregulation contributes significantly to tumor initiation and progression, making ncRNAs valuable biomarkers for early detection, prognosis, and disease monitoring. Looking ahead, future research should focus on improving the clinical translation of ncRNA-based diagnostics and therapeutics. Challenges include advanced delivery systems, off-target effects, and standardization of detection methods. Advances in nanotechnology and precision medicine are expected to enhance the specificity and efficacy of ncRNA-targeted therapies.

Future research in hereditary breast cancer is expected to benefit from integrative multi-omics approaches, including genomics, transcriptomics, and epigenomics, which may provide a more comprehensive understanding of tumor heterogeneity and non-coding RNA regulatory networks. In addition, artificial intelligence and machine learning-based models hold promise for improving individualized risk prediction by integrating germline variants and clinical features, ultimately advancing precision medicine strategies for hereditary breast cancer.

## Data Availability

No datasets were generated or analysed during the current study.

## Abbreviations

ADCs: Antibody-drug conjugates
APOBEC3: Apolipoprotein B mRNA-editing enzyme catalytic polypeptide-like 3
circRNAs: Circular RNAs
GWAS: Genome-wide association studies
lncRNAs: Long non-coding RNAs
miRNAs: MicroRNAs
mRNA: Messenger RNA
ncRNAs: Non-coding RNAs
P/LP: Pathogenic or likely pathogenic
piRNAs: Piwi-interacting RNAs
PRS: Polygenic risk scores
TNBC: Triple-negative breast cancer

## References

[1] Breast cancer: World Health Organization. 2026. Available from: https://www.who.int/news-room/fact-sheets/detail/breast-cancer. [updated 2026-04-16; Cited 2026 05–16].

[2] Carvalho E, Canberk S, Schmitt F, Vale N. Molecular subtypes and mechanisms of breast cancer: precision medicine approaches for targeted therapies. Cancers. 2025;17(7):1102. 10.3390/cancers17071102. PMC11987866 40227634

[3] Grabinski VF, Brawley OW. Disparities in Breast Cancer. Obstet Gynecol Clin N Am. 2022;49(1):149–65. 10.1016/j.ogc.2021.11.010 35168767

[4] Saka AH, Giaquinto AN, McCullough LE, Tossas KY, Star J, Jemal A, et al. Cancer statistics for African American and Black people, 2025. Cancer J Clin. 2025;75(2):111–40. 10.3322/caac.21874 PMC11929131 39976243

[5] Xu H, Xu B. Breast cancer: Epidemiology, risk factors and screening. Chin J cancer Res = Chung-kuo yen cheng yen chiu. 2023;35(6):565–83. 10.21147/j.issn.1000-9604.2023.06.02 PMC10774137 38204449

[6] Morimoto T, Nagao T, Okazaki K, Kira M, Nakagawa Y, Tangoku A. Current status of breast cancer screening in the world. Breast cancer (Tokyo Japan). 2009;16(1):2–9. 10.1007/s12282-008-0077-5 18841333

[7] Omidiji OA, Campbell PC, Irurhe NK, Atalabi OM, Toyobo OO. Breast cancer screening in a resource poor country: Ultrasound versus mammography. Ghana Med J. 2017;51(1):6–12. 10.4314/gmj.v51i1.2 PMC5611948 28959066

[8] Johnson KS, Conant EF, Soo MS. Molecular Subtypes of Breast Cancer: A Review for Breast Radiologists. J breast imaging. 2021;3(1):12–24. 10.1093/jbi/wbaa110 38424845

[9] Zhang L, Huang Y, Feng Z, Wang X, Li H, Song F, et al. Comparison of breast cancer risk factors among molecular subtypes: A case-only study. Cancer Med. 2019;8(4):1882–92. 10.1002/cam4.2012 PMC6488156 30761775

[10] Jahangiri L. Genetic Aspects of Breast Cancer: an Update. Curr Breast Cancer Rep. 2026;18(1):11.

[11] Antoniou A, Pharoah PD, Narod S, Risch HA, Eyfjord JE, Hopper JL, et al. Average risks of breast and ovarian cancer associated with BRCA1 or BRCA2 mutations detected in case Series unselected for family history: a combined analysis of 22 studies. Am J Hum Genet. 2003;72(5):1117–30. 10.1086/375033 PMC1180265 12677558

[12] Tanha HM, Law MH, Ingold N, Olsen CM, Pandeya N, Milne RL, et al. Performance of different polygenic risk scores for breast cancer risk prediction: in-depth evaluations across large UK and Australian cohorts. Eur J Hum genetics: EJHG. 2026;34(2):278–87. 10.1038/s41431-025-02003-8 PMC12858958 41526671

[13] Hereditary Cancer Awareness Week. Available from: https://ovarian.org.uk/get-involved/campaigns/hereditary-cancer-awareness-week/. [cited 2026 05-16].

[14] Godet I, Gilkes DM. BRCA1 and BRCA2 mutations and treatment strategies for breast cancer. Integr Cancer Sci Ther. 2017;4(1):10.15761/ICST.1000228. 10.15761/ICST.1000228. PMC5505673 28706734

[15] Peleg Hasson S, Menes T, Sonnenblick A. Comparison of Patient Susceptibility Genes Across Breast Cancer: Implications for Prognosis and Therapeutic Outcomes. Pharmacogenomics personalized Med. 2020;13:227–38. 10.2147/PGPM.S233485 PMC7394592 32801835

[16] Dorling L, Carvalho S, Allen J, González-Neira A, Luccarini C, Wahlström C, et al. Breast Cancer Risk Genes - Association Analysis in More than 113,000 Women. N Engl J Med. 2021;384(5):428–39. 10.1056/NEJMoa1913948 PMC7611105 33471991

[17] Pal M, Das D, Pandey M. Understanding genetic variations associated with familial breast cancer. World J Surg Oncol. 2024;22(1):271. 10.1186/s12957-024-03553-9 PMC11465949 39390525

[18] Fan X, Wynn J, Shang N, Liu C, Fedotov A, Hallquist MLG, et al. Penetrance of breast cancer susceptibility genes from the eMERGE III network. JNCI Cancer Spectr. 2021;5(4):pkab044. 10.1093/jncics/pkab044. PMC8346699 34377931

[19] Antoniou AC, Casadei S, Heikkinen T, Barrowdale D, Pylkäs K, Roberts J, et al. Breast-cancer risk in families with mutations in PALB2. N Engl J Med. 2014;371(6):497–506. 10.1056/NEJMoa1400382 PMC4157599 25099575

[20] Boonen R, Wiegant WW, Celosse N, Vroling B, Heijl S, Kote-Jarai Z, et al. Functional Analysis Identifies Damaging CHEK2 Missense Variants Associated with Increased Cancer Risk. Cancer Res. 2022;82(4):615–31. 10.1158/0008-5472.CAN-21-1845 PMC9359737 34903604

[21] McDuff SGR, Bellon JR, Shannon KM, Gadd MA, Dunn S, Rosenstein BS, et al. ATM Variants in Breast Cancer: Implications for Breast Radiation Therapy Treatment Recommendations. Int J Radiat Oncol Biol Phys. 2021;110(5):1373–82. 10.1016/j.ijrobp.2021.01.045 33545302

[22] Rizzolo P, Silvestri V, Falchetti M, Ottini L. Inherited and acquired alterations in development of breast cancer. application Clin Genet. 2011;4:145–58. 10.2147/TACG.S13226 PMC3681186 23776375

[23] Xiong X, Zheng LW, Ding Y, Chen YF, Cai YW, Wang LP, et al. Breast cancer: pathogenesis and treatments. Signal Transduct Target therapy. 2025;10(1):49. 10.1038/s41392-024-02108-4 PMC11836418 39966355

[24] Mei W, Faraj Tabrizi S, Godina C, Lovisa AF, Isaksson K, Jernström H, et al. A commonly inherited human PCSK9 germline variant drives breast cancer metastasis via LRP1 receptor. Cell. 2025;188(2):371–e8928. 10.1016/j.cell.2024.11.009 PMC11770377 39657676

[25] Li CM, Cordes A, Oliphant MUJ, Quinn SA, Thomas M, Selfors LM, et al. Brca1 haploinsufficiency promotes early tumor onset and epigenetic alterations in a mouse model of hereditary breast cancer. Nat Genet. 2024;56(12):2763–75. 10.1038/s41588-024-01958-6 39528827

[26] Yao S, Wei L, Hu Q, Liu S, Manojlovic Z, Fiorica PN, et al. Mutational landscape of triple-negative breast cancer in African American women. Nat Genet. 2025;57(9):2166–76. 10.1038/s41588-025-02322-y PMC12425817 40858906

[27] Jia G, Chen Z, Ping J, Cai Q, Tao R, Li C, et al. Refining breast cancer genetic risk and biology through multi-ancestry fine-mapping analyses of 192 risk regions. Nat Genet. 2025;57(1):80–7. 10.1038/s41588-024-02031-y PMC12184877 39753771

[28] Zhang Y, Lindström S, Kraft P, Liu Y. Genetic risk, health-associated lifestyle, and risk of early-onset total cancer and breast cancer. J Natl Cancer Inst. 2025;117(1):40–8. 10.1093/jnci/djae208 PMC11717420 39189966

[29] Roberts E, Howell S, Evans DG. Polygenic risk scores and breast cancer risk prediction. Breast (Edinburgh Scotland). 2023;67:71–7. 10.1016/j.breast.2023.01.003 PMC9982311 36646003

[30] Nguyen HHK, Le HM, Le TQ, Dinh NTQ, Nguyen TT, Nguyen PA, et al. Polygenic risk scores: Navigating the future of precision medicine through economic, ethical, and scientific advancements. iScience. 2026;29(1):114375. 10.1016/j.isci.2025.114375 PMC12800428 41541687

[31] Mavaddat N, Michailidou K, Dennis J, Lush M, Fachal L, Lee A, et al. Polygenic Risk Scores for Prediction of Breast Cancer and Breast Cancer Subtypes. Am J Hum Genet. 2019;104(1):21–34. 10.1016/j.ajhg.2018.11.002 PMC6323553 30554720

[32] Ho WK, Tan MM, Mavaddat N, Tai MC, Mariapun S, Li J, et al. European polygenic risk score for prediction of breast cancer shows similar performance in Asian women. Nat Commun. 2020;11(1):3833. 10.1038/s41467-020-17680-w PMC7395776 32737321

[33] Garcia-Closas M, Gunsoy NB, Chatterjee N. Combined associations of genetic and environmental risk factors: implications for prevention of breast cancer. J Natl Cancer Inst. 2014;106(11):dju305. 10.1093/jnci/dju305. PMC4271030 25392194

[34] Wu GY, Xiao MZ, Hao WC, Yang ZS, Liu XR, Xu DS, et al. Drug resistance in breast cancer: Mechanisms and strategies for management. Drug Resist updates: reviews commentaries Antimicrob anticancer Chemother. 2025;83:101288. 10.1016/j.drup.2025.101288 40795794

[35] Gupta A, Gazzo A, Selenica P, Safonov A, Pareja F, da Silva EM, et al. APOBEC3 mutagenesis drives therapy resistance in breast cancer. Nat Genet. 2025;57(6):1452–62. 10.1038/s41588-025-02187-1 PMC12165862 40379787

[36] Nolan E, Lindeman GJ, Visvader JE. Deciphering breast cancer: from biology to the clinic. Cell. 2023;186(8):1708–28. 10.1016/j.cell.2023.01.040 36931265

[37] Daly MB, Pal T, Maxwell KN, Churpek J, Kohlmann W, AlHilli Z, et al. NCCN Guidelines^®^ Insights: Genetic/Familial High-Risk Assessment: Breast, Ovarian, and Pancreatic, Version 2.2024. J Natl Compr Cancer Network: JNCCN. 2023;21(10):1000–10. 10.6004/jnccn.2023.0051 37856201

[38] Bedrosian I, Somerfield MR, Achatz MI, Boughey JC, Curigliano G, Friedman S, et al. Germline Testing in Patients With Breast Cancer: ASCO-Society of Surgical Oncology Guideline. J Clin oncology: official J Am Soc Clin Oncol. 2024;42(5):584–604. 10.1200/JCO.23.02225 38175972

[39] Sessa C, Balmaña J, Bober SL, Cardoso MJ, Colombo N, Curigliano G, et al. Risk reduction and screening of cancer in hereditary breast-ovarian cancer syndromes: ESMO Clinical Practice Guideline. Annals oncology: official J Eur Soc Med Oncol. 2023;34(1):33–47. 10.1016/j.annonc.2022.10.004 36307055

[40] Guha D, Mondal J, Nandy A, Biswas S, Bagchi A. Interplay of circular RNAs in gastric cancer - a systematic review. Front Syst biology. 2024;4:1497510. 10.3389/fsysb.2024.1497510 PMC12341982 40809111

[41] Beňačka R, Szabóová D, Guľašová Z, Hertelyová Z, Radoňak J. Non-coding RNAs in human cancer and other diseases: overview of the diagnostic potential. Int J Mol Sci. 2023;24(22):16213. 10.3390/ijms242216213. PMC10671391 38003403

[42] Tanic M, Yanowski K, Gómez-López G, Rodriguez-Pinilla MS, Marquez-Rodas I, Osorio A, et al. MicroRNA expression signatures for the prediction of BRCA1/2 mutation-associated hereditary breast cancer in paraffin-embedded formalin-fixed breast tumors. Int J Cancer. 2015;136(3):593–602. 10.1002/ijc.29021 24917463

[43] Murria Estal R, Palanca Suela S, de Juan Jiménez I, Egoavil Rojas C, García-Casado Z, Juan Fita MJ, et al. MicroRNA signatures in hereditary breast cancer. Breast Cancer Res Treat. 2013;142(1):19–30. 10.1007/s10549-013-2723-7 24129975

[44] Pinto R, De Summa S, Pilato B, Tommasi S. DNA methylation and miRNAs regulation in hereditary breast cancer: epigenetic changes, players in transcriptional and post- transcriptional regulation in hereditary breast cancer. Curr Mol Med. 2014;14(1):45–57. 10.2174/1566524013666131203101405 24295492

[45] Tanic M, Yanowsky K, Rodriguez-Antona C, Andrés R, Márquez-Rodas I, Osorio A, et al. Deregulated miRNAs in hereditary breast cancer revealed a role for miR-30c in regulating KRAS oncogene. PLoS ONE. 2012;7(6):e38847. 10.1371/journal.pone.0038847 PMC3372467 22701724

[46] Landeros N, Gonzalez-Hormazabal P, Pérez-Moreno P, Tapia JC, Jara L. A single variant in pri-miRNA-155 associated with susceptibility to hereditary breast cancer promotes aggressiveness in breast cancer cells. Int J Mol Sci. 2022;23(23):15418. 10.3390/ijms232315418. PMC9735695 36499743

[47] Cantile M, Di Bonito M, Cerrone M, Collina F, De Laurentiis M, Botti G. Long non-coding RNA HOTAIR in breast cancer therapy. Cancers. 2020;12(5):1197. 10.3390/cancers12051197. PMC7281113 32397382

[48] Yue X, Wu WY, Dong M, Guo M. LncRNA MALAT1 promotes breast cancer progression and doxorubicin resistance via regulating miR-570-3p. Biomedical J. 2021;44(6 Suppl 2):S296–304. 10.1016/j.bj.2020.11.002 PMC9068547 35410813

[49] Wang Y, Zhang Y, Hu K, Qiu J, Hu Y, Zhou M, et al. Elevated long noncoding RNA MALAT-1 expression is predictive of poor prognosis in patients with breast cancer: a meta-analysis. Biosci Rep. 2020;40(8):BSR20200215. 10.1042/BSR20200215. PMC7419804 32700729

[50] Zhou Y, Zhong Y, Wang Y, Zhang X, Batista DL, Gejman R, et al. Activation of p53 by MEG3 non-coding RNA. J Biol Chem. 2007;282(34):24731–42. 10.1074/jbc.M702029200 17569660

[51] Zhang JJ, Guo SH, Jia BQ. Down-regulation of long non-coding RNA MEG3 serves as an unfavorable risk factor for survival of patients with breast cancer. Eur Rev Med Pharmacol Sci. 2016;20(24):5143–7. 28051255

[52] Shin VY, Chen J, Cheuk IW, Siu MT, Ho CW, Wang X, et al. Long non-coding RNA NEAT1 confers oncogenic role in triple-negative breast cancer through modulating chemoresistance and cancer stemness. Cell Death Dis. 2019;10(4):270. 10.1038/s41419-019-1513-5 PMC6426882 30894512

[53] Li S, Hao J, Hong Y, Mai J, Huang W. Long Non-Coding RNA NEAT1 Promotes the Proliferation, Migration, and Metastasis of Human Breast-Cancer Cells by Inhibiting miR-146b-5p Expression. Cancer Manage Res. 2020;12:6091–101. 10.2147/CMAR.S252295 PMC7382757 32801860

[54] Fang H, Xie J, Zhang M, Zhao Z, Wan Y, Yao Y. miRNA-21 promotes proliferation and invasion of triple-negative breast cancer cells through targeting PTEN. Am J translational Res. 2017;9(3):953–61. PMC5375989 28386324

[55] Chen J, Wang X. MicroRNA-21 in breast cancer: diagnostic and prognostic potential. Clinical & translational oncology: official. publication Federation Span Oncol Soc Natl Cancer Inst Mexico. 2014;16(3):225–33. 10.1007/s12094-013-1132-z 24248894

[56] Wang J, Wang Q, Guan Y, Sun Y, Wang X, Lively K, et al. Breast cancer cell-derived microRNA-155 suppresses tumor progression via enhancing immune cell recruitment and antitumor function. J Clin Investig. 2022;132(19):e157248. 10.1172/JCI157248. PMC9525116 35925680

[57] Yahya SMM, Nabih HK, Elsayed GH, Mohamed SIA, Elfiky AM, Salem SM. Restoring microRNA-34a overcomes acquired drug resistance and disease progression in human breast cancer cell lines via suppressing the ABCC1 gene. Breast Cancer Res Treat. 2024;204(1):133–49. 10.1007/s10549-023-07170-0 PMC10806220 38057687

[58] Korpal M, Lee ES, Hu G, Kang Y. The miR-200 family inhibits epithelial-mesenchymal transition and cancer cell migration by direct targeting of E-cadherin transcriptional repressors ZEB1 and ZEB2. J Biol Chem. 2008;283(22):14910–4. 10.1074/jbc.C800074200 PMC3258899 18411277

[59] Thi Chung Duong T, Nguyen THN, Thi Ngoc Nguyen T, Huynh LH, Ngo HP, Thi Nguyen H. Diagnostic and prognostic value of miR-200 family in breast cancer: A meta-analysis and systematic review. Cancer Epidemiol. 2022;77:102097. 10.1016/j.canep.2022.102097 35030348

[60] Zheng Q, Bao C, Guo W, Li S, Chen J, Chen B, et al. Circular RNA profiling reveals an abundant circHIPK3 that regulates cell growth by sponging multiple miRNAs. Nat Commun. 2016;7:11215. 10.1038/ncomms11215 PMC4823868 27050392

[61] Bahadoran E, Mohammadi D, Jalilvand M, Moghbelinejad S. Evaluation of CircHIPK3 biomarker potential in breast cancer. Practical Lab Med. 2025;45:e00470. 10.1016/j.plabm.2025.e00470 PMC11984561 40226123

[62] Yang W, Yang X, Wang X, Gu J, Zhou D, Wang Y, et al. Silencing CDR1as enhances the sensitivity of breast cancer cells to drug resistance by acting as a miR-7 sponge to down-regulate REGγ. J Cell Mol Med. 2019;23(8):4921–32. 10.1111/jcmm.14305 PMC6652952 31245927

[63] Yuan C, Xu Y, Zhou L, Peng J, Sha R, Lin Y, et al. Value of CDR1-AS as a predictive and prognostic biomarker for patients with breast cancer receiving neoadjuvant chemotherapy in a prospective Chinese cohort. Eur J Med Res. 2024;29(1):454. 10.1186/s40001-024-02015-y PMC11389417 39261936

[64] Liang HF, Zhang XZ, Liu BG, Jia GT, Li WL. Circular RNA circ-ABCB10 promotes breast cancer proliferation and progression through sponging miR-1271. Am J cancer Res. 2017;7(7):1566–76. PMC5523036 28744405

[65] İlhan B, Ender Ş, Kılıç B, Üçüncü M, Serilmez M, Tilgen Yasasever C, et al. The Diagnostic Value of circFBXW7, circABCB10, and circ0103552 Levels in Breast Cancer. Curr Issues Mol Biol. 2024;46(12):14381–93. 10.3390/cimb46120862 PMC11674701 39727990

[66] Chen D, Zeng S, Qiu H, Yang M, Lin X, Lv X, et al. Circ-FOXO3 inhibits triple-negative breast cancer growth and metastasis via regulating WHSC1-H3K36me2-Zeb2 axis. Cell Signal. 2024;117:111079. 10.1016/j.cellsig.2024.111079 38341124

[67] Öner Ç, Köser F, Çolak E. The association of piR-651 and piR-823 on metastatic and invasive characteristics of triple negative breast cancer cells. Nucleosides Nucleotides Nucleic Acids. 2026;45(1):1–17. 10.1080/15257770.2024.2437037 39630699

[68] Liu T, Wang J, Sun L, Li M, He X, Jiang J, et al. Piwi-interacting RNA-651 promotes cell proliferation and migration and inhibits apoptosis in breast cancer by facilitating DNMT1-mediated PTEN promoter methylation. Cell cycle (Georgetown Tex). 2021;20(16):1603–16. 10.1080/15384101.2021.1956090 PMC8409782 34313525

[69] Ding X, Li Y, Lü J, Zhao Q, Guo Y, Lu Z, et al. piRNA-823 Is Involved in Cancer Stem Cell Regulation Through Altering DNA Methylation in Association With Luminal Breast Cancer. Front cell Dev biology. 2021;9:641052. 10.3389/fcell.2021.641052 PMC8005588 33791297

[70] Tan L, Mai D, Zhang B, Jiang X, Zhang J, Bai R, et al. PIWI-interacting RNA-36712 restrains breast cancer progression and chemoresistance by interaction with SEPW1 pseudogene SEPW1P RNA. Mol Cancer. 2019;18(1):9. 10.1186/s12943-019-0940-3 PMC6330501 30636640

[71] Nadhan R, Isidoro C, Song YS, Dhanasekaran DN. Signaling by LncRNAs: structure, cellular homeostasis, and disease pathology. Cells. 2022;11(16):2517. 10.3390/cells11162517. PMC9406440 36010595

[72] Solaimani M, Hosseinzadeh S, Abasi M. Non-coding RNAs, a double-edged sword in breast cancer prognosis. Cancer Cell Int. 2025;25(1):123. 10.1186/s12935-025-03679-0 PMC11959806 40170036

[73] Bhattacharjee R, Prabhakar N, Kumar L, Bhattacharjee A, Kar S, Malik S, et al. Crosstalk between long noncoding RNA and microRNA in Cancer. Cell Oncol (Dordrecht Netherlands). 2023;46(4):885–908. 10.1007/s13402-023-00806-9 PMC10356678 37245177

[74] Xiao B, Zhang W, Chen L, Hang J, Wang L, Zhang R, et al. Analysis of the miRNA-mRNA-lncRNA network in human estrogen receptor-positive and estrogen receptor-negative breast cancer based on TCGA data. Gene. 2018;658:28–35. 10.1016/j.gene.2018.03.011 29518546

[75] Shinde SS, Ahmed S, Malik JA, Hani U, Khanam A, Ashraf Bhat F, et al. Therapeutic delivery of tumor suppressor miRNAs for breast cancer treatment. Biology. 2023;12(3):467. 10.3390/biology12030467. PMC10045434 36979159

[76] O’Brien J, Hayder H, Zayed Y, Peng C. Overview of MicroRNA Biogenesis, Mechanisms of Actions, and Circulation. Front Endocrinol. 2018;9:402. 10.3389/fendo.2018.00402 PMC6085463 30123182

[77] Chang BP, Wang DS, Xing JW, Yang SH, Chu Q, Yu SY. miR-200c inhibits metastasis of breast cancer cells by targeting HMGB1. Journal of Huazhong University of Science and Technology Medical sciences = Hua zhong ke ji da xue xue bao Yi xue Ying De wen ban = Huazhong keji daxue xuebao. Yixue Yingdewen ban. 2014;34(2):201–6. 10.1007/s11596-014-1259-3 24710933

[78] Thammaiah CK, Jayaram S. Role of let-7 family microRNA in breast cancer. Non-coding RNA Res. 2016;1(1):77–82. 10.1016/j.ncrna.2016.10.003 PMC6096426 30159414

[79] Zhang Y, Yan LX, Wu QN, Du ZM, Chen J, Liao DZ, et al. miR-125b is methylated and functions as a tumor suppressor by regulating the ETS1 proto-oncogene in human invasive breast cancer. Cancer Res. 2011;71(10):3552–62. 10.1158/0008-5472.CAN-10-2435 21444677

[80] Hossainzadeh S, Ranji N, Naderi Sohi A, Najafi F. Silibinin encapsulation in polymersome: A promising anticancer nanoparticle for inducing apoptosis and decreasing the expression level of miR-125b/miR-182 in human breast cancer cells. J Cell Physiol. 2019;234(12):22285–98. 10.1002/jcp.28795 31073992

[81] Meng S, Zhou H, Feng Z, Xu Z, Tang Y, Li P, et al. CircRNA: functions and properties of a novel potential biomarker for cancer. Mol Cancer. 2017;16(1):94. 10.1186/s12943-017-0663-2 PMC5440908 28535767

[82] Yi Q, Feng J, Lan W, Shi H, Sun W, Sun W. CircRNA and lncRNA-encoded peptide in diseases, an update review. Mol Cancer. 2024;23(1):214. 10.1186/s12943-024-02131-7 PMC11441268 39343883

[83] Du WW, Fang L, Yang W, Wu N, Awan FM, Yang Z, et al. Induction of tumor apoptosis through a circular RNA enhancing Foxo3 activity. Cell Death Differ. 2017;24(2):357–70. 10.1038/cdd.2016.133 PMC5299715 27886165

[84] Yang Q, Wu J, Su Y. Emerging roles of PIWI-interacting RNAs in cancer molecular diagnostics and therapeutics: a molecular biosciences perspective. Front Mol Biosci. 2026;13:1775335. 10.3389/fmolb.2026.1775335 PMC13095571 42022287

[85] Wu Z, Yu X, Zhang S, He Y, Guo W. Novel roles of PIWI proteins and PIWI-interacting RNAs in human health and diseases. Cell communication signaling: CCS. 2023;21(1):343. 10.1186/s12964-023-01368-x PMC10685540 38031146

[86] Eapen JV, George P, Thomas S, Antony J. Integrative strategies in breast cancer therapy: Conventional approaches, emerging advances, and future challenges. ASPET Discovery. 2025;1:100012.

[87] Kreutzfeldt J, Rozeboom B, Dey N, De P. The trastuzumab era: current and upcoming targeted HER2 + breast cancer therapies. Am J cancer Res. 2020;10(4):1045–67. PMC7191090 32368385

[88] Tufail M, Cui J, Wu C. Breast cancer: molecular mechanisms of underlying resistance and therapeutic approaches. Am J cancer Res. 2022;12(7):2920–49. PMC9360230 35968356

[89] Li R, Ji Y, Ye R, Tang G, Wang W, Chen C, et al. Potential therapies for non-coding RNAs in breast cancer. Front Oncol. 2024;14:1452666. 10.3389/fonc.2024.1452666 PMC11449682 39372872

[90] Beňačka R, Szabóová D, Guľašová Z, Hertelyová Z. Non-coding RNAs in breast cancer: diagnostic and therapeutic implications. Int J Mol Sci. 2024;26(1):127. 10.3390/ijms26010127. PMC11719911 39795985

[91] Menon A, Abd-Aziz N, Khalid K, Poh CL, Naidu R. miRNA: A Promising Therapeutic Target in Cancer. Int J Mol Sci. 2022;23:19. 10.3390/ijms231911502 PMC9569513 36232799

[92] Rejili M. Clinical advances and challenges of antibody-mediated targeted drug delivery in breast cancer therapeutics. Discover Oncol. 2026;17(1):377. 10.1007/s12672-026-04492-5. PMC12953841 41621018

[93] Dvir K, Giordano S, Leone JP. Immunotherapy in breast cancer. Int J Mol Sci. 2024;25(14):7517. 10.3390/ijms25147517. PMC11276856 39062758

[94] Papalexis P, Georgakopoulou VE, Drossos PV, Thymara E, Nonni A, Lazaris AC, et al. Precision medicine in breast cancer (Review). Mol Clin Oncol. 2024;21(5):78. 10.3892/mco.2024.2776 PMC11375768 39246849

[95] Wang Y, Minden A. Current molecular combination therapies used for the treatment of breast cancer. Int J Mol Sci. 2022;23(19):11046. 10.3390/ijms231911046. PMC9569555 36232349

[96] Samad MA, Ahmad I, Zughaibi TA, Suhail M, Zaidi SK, Al-Abbasi FA, et al. Nanotechnology-based drug delivery for breast cancer treatment: Current applications and future directions. Eur J Med Chem Rep. 2025;14:100268.

[97] Izzo D, Ascione L, Guidi L, Marsicano RM, Koukoutzeli C, Trapani D, et al. Innovative payloads for ADCs in cancer treatment: moving beyond the selective delivery of chemotherapy. Therapeutic Adv Med Oncol. 2025;17:17588359241309461. 10.1177/17588359241309461 PMC11694294 39759830

[98] Paz-Manrique R, Pinto JA, Gomez Moreno HL. Antibody-Drug Conjugates (ADCs) for Breast Cancer Therapeutic Landscape: Concept and Mechanisms of Action. Hematol Oncol Stem Cell Ther. 2025;18(4):133–9. 10.4103/hemoncstem.HEMONCSTEM-D-24-00042 41630133

[99] Jamalinia M, Weiskirchen R. Advances in personalized medicine: translating genomic insights into targeted therapies for cancer treatment. Annals translational Med. 2025;13(2):18. 10.21037/atm-25-34 PMC12106117 40438512

[100] Menezes MCS, Raheem F, Mina L, Ernst B, Batalini F. PARP inhibitors for breast cancer: germline BRCA1/2 and beyond. Cancers. 2022;14(17):4332. 10.3390/cancers14174332. PMC9454726 36077867

[101] Alharbi S, Qasem FF, Talebi MT, Omran NE, Hamoudi R, Harati R. Immunotherapy approaches for the treatment of triple-negative breast cancer. Cancers. 2026;18(3):464. 10.3390/cancers18030464. PMC12897434 41681936

[102] Lv Y, Li W, Liao W, Jiang H, Liu Y, Cao J, et al. Nano-Drug Delivery Systems Based on Natural Products. Int J Nanomed. 2024;19:541–69. 10.2147/IJN.S443692 PMC10802180 38260243

[103] Islam S, Ahmed MMS, Islam MA, Hossain N, Chowdhury MA. Advances in nanoparticles in targeted drug delivery–A review. Results Surf Interfaces. 2025;19:100529.

[104] Delou JMA, Souza ASO, Souza LCM, Borges HL. Highlights in resistance mechanism pathways for combination therapy. Cells. 2019;8(9):1013. 10.3390/cells8091013. PMC6770082 31480389

